# Scientific QUEERies: an interview with Scott Cocker and Kyle Shanebeck on improving LGBTQ2S+ visibility in STEM

**DOI:** 10.1038/s42003-021-02811-w

**Published:** 2021-11-18

## Abstract

In recognition of LGBTQ+ STEM Day on November 18th, we celebrate the achievements of queer researchers and their efforts to improve representation in STEM. Scott Cocker and Kyle Shanebeck are PhD students at the University of Alberta and co-founders of Scientific QUEERies, a biweekly seminar series that provides a platform for queer STEM professionals to share their achievements and personal stories. In this Q&A, we asked Scott and Kyle about their own research experiences, what it means to be queer in STEM, and the importance of initiatives like Scientific QUEERies.

Scott Cocker (he/him) completed his undergraduate degree in Geology and Physical Geography at the University of Edinburgh before moving to Canada to pursue his graduate work in quaternary paleoecology. He received his master’s degree at Brock University, Ontario, where he focused on further developing methods to track the presence of megaherbivores, such as the woolly mammoth, in ice age east Beringia (Alaska and Yukon Territory) in situations where bone samples are not available. His current PhD work with Dr. Duane Froese at the University of Alberta revolves around ancient DNA preserved in permafrost, with a focus on long-term records of arctic ground squirrels (*Urocitellus parryii*).

**Figure:**
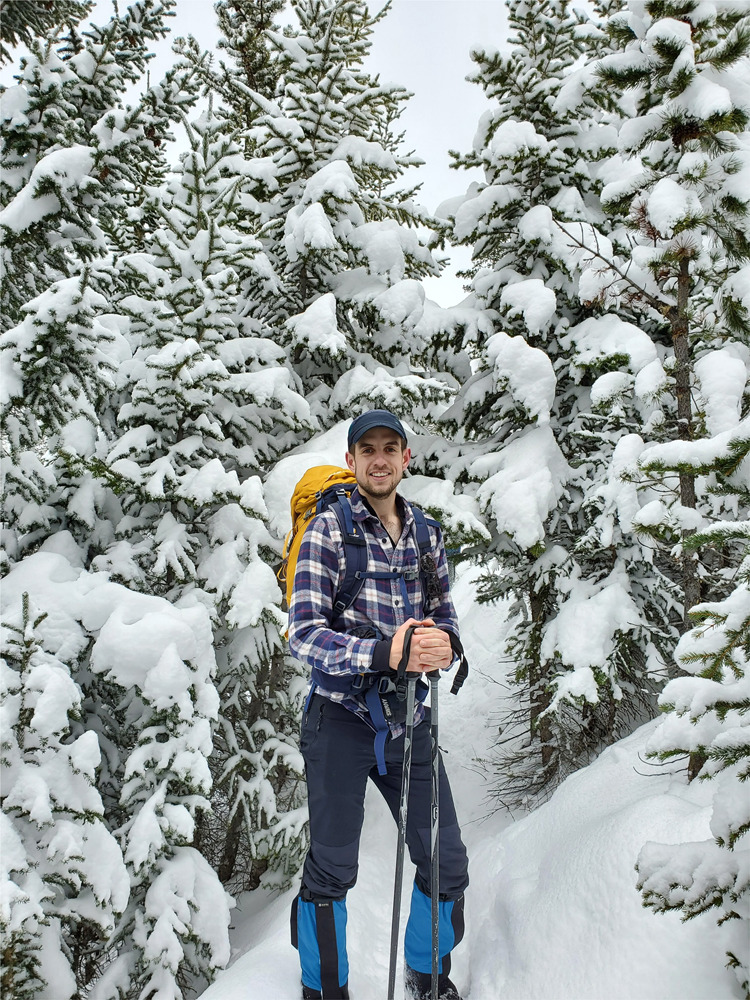
Scott Cocker

Kyle Shanebeck (he/they) grew up in southern California and received his undergraduate degree from Biola University. He received his master’s in Ecology at the Universität Bremen in Germany, where he investigated the intestinal parasites of otters and seals, specifically related species of *Corynosoma sp*. (*Acanthocephala*), and differences in their site selection and reproduction. During this time, he also established collaborations with the Marine Wildlife Care and Research Center, California Department of Fish and Wildlife and the Marine Mammal Office, US Fish and Wildlife Service, Alaska Region. Kyle moved from Germany to Canada to begin his PhD at the University of Alberta, where he investigates the energetic effects of sublethal parasite infections in aquatic mammals like the river otter (*Lontra canadensis*) and mink (*Neogale vison*).

**Figure:**
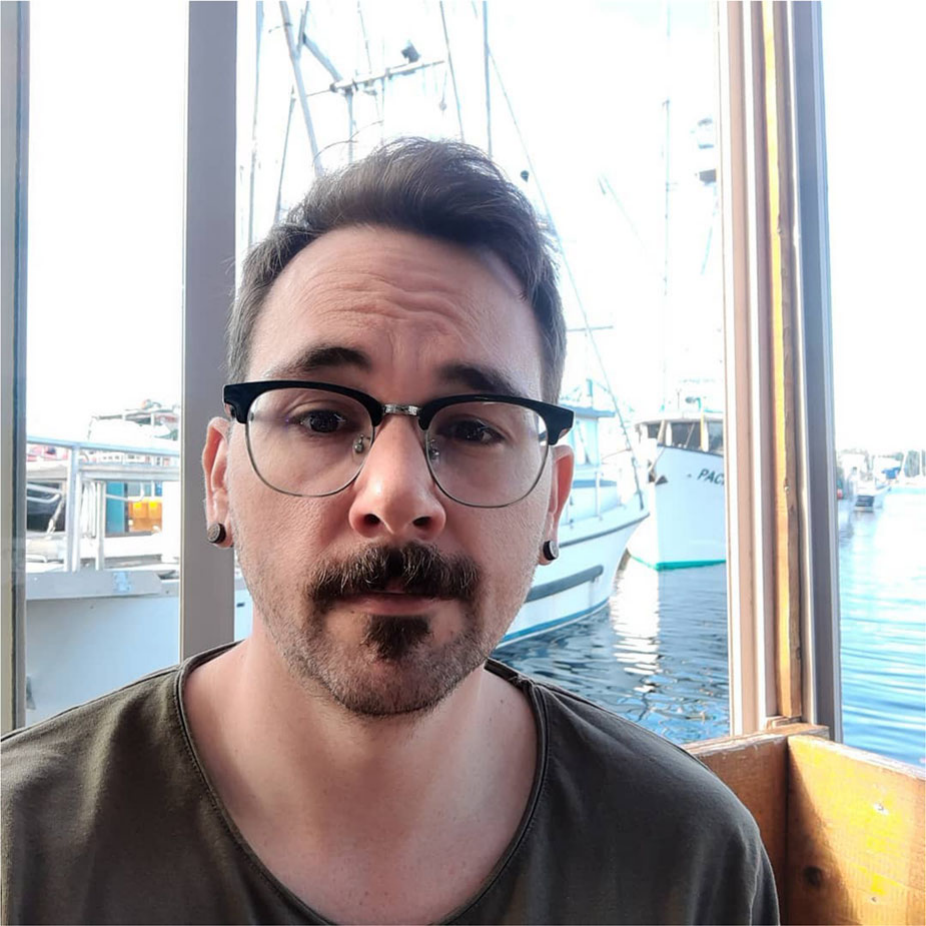
Kyle Shanebeck

**What does it mean to you, to be queer in STEM?**

**Scott Cocker (SC):** For the longest time, as an invisible minority, being queer in STEM meant being alone. It meant trying to decide if “coming out” would affect graduate opportunities, where I could study, and career aspirations. I’m so glad to say that this narrative has changed for me, for the better. Given I have the safety to do so, being a visible member of the LGBTQ2S+ community has for the first time, been beneficial for making connections and embracing my queer self. I have been extremely fortunate to land myself in a department that actively encourages me to be vocal and provides me, and others, the platform to discuss pressing issues relating to equity, diversity and inclusion.

**Kyle Shanebeck (KS):** It means being mostly invisible. I have not found many, if any, opportunities for my identity as a queer person to have space in my life as a scientist. It also made my path to success in academia much harder due to systemic inequalities. Like many queer people, I had to support myself alone, through poverty, while trying to succeed in my chosen career. I did not have the resources to go to a research university, to pay for expensive GRE training courses, or rely on family to support me while I volunteered full time in a non-profit or research group to gain “experience.” I cannot apply for positions or programs that work in countries where being queer is illegal, or where I would be required to move to a rural community which often are less safe for queer people.

I have experienced bias in workspaces dominated both by cis-men, who viewed me as incompetent for my femininity, and by cis-woman, who treated me either with tokenism or exclusion for my masculinity. A former boss, for example, told me I was hired because she “loves gay guys” since they are “non-threatening” and love to gossip. I have been marginalized, infantilized, and fired for my gender and sexual identity. It is a constant struggle to decide when to disclose my identity, as I have a constant fear of rejection and bias; particularly in job or scholarship applications where I know my identity could either be a benefit or an immediate detriment (and it feels like a gamble either way).

**What do you think still needs to change for LGBTQ2S**+ **researchers in STEM?**

**SC:** Despite making steps in the right direction, it is still apparent that STEM institutions are simply looking to tick the “diversity” box and move on. We need more than just visibility. LGBTQ2S+ researchers, staff, and students need the ability to build strong and resilient communities to normalize our place in society. I think this is particularly important for queer students in order to provide them the opportunity to meet people like them and the allies willing to support them. Little things can make an absolute difference. On your lab website, make it clear that you support and encourage diversity. Be blatant, write a standalone statement that outlines the values of you, as a PI, and your group. This can be crucial in decision making for graduate school, I can speak from experience on this.

**KS:** Visibility, as well as knowledge of the systemic barriers that young queer people must overcome to even get into science. There is a myth that we work within a meritocracy in STEM, but the reality is that cis, straight, white people are more likely to have greater access to the resume-building activities that make them more “qualified” just because of their average social, educational and health situations. LGBTQ2S+ people are more likely to live in poverty (often because of rejection by their families), to experience serious mental and physical health issues, and to be passed over for jobs/scholarships/raises just because of their identity, which limits their professional opportunities. We need more emphasis on individual experiences and to reject the idea that holding everyone to the same standard of resume length and content is “equality.” I also think that STEM needs to understand that diversity is important not just because it is morally good (or politically correct), but because it makes institutions better. Queer people, people of color, people with disabilities, and other minorities bring lived experience and ways of thinking that are unique, which strengthens a team. We are resilient, hard-working, and creative because you must be to overcome inequalities. We are valuable not because we fulfill a diversity quota, but because we have perspectives and experience that cis/straight people do not.

**What inspired you to create Scientific QUEERies? Do you have any advice for trainees who might want to start similar programs at their institutions?**

**SC:** We decided to create Scientific QUEERies simply because there were no other initiatives close to us that we could directly relate to. Kyle and I have very different experiences from each other. Growing up in families with conflicting value systems in two different countries. However, there was a clear combined yearning for representation of LGBTQ2S+ individuals in our university community, and we wanted to provide the platform to do just that. I don’t think either of us realized, initially, how far reaching we could make Scientific QUEERies, but here we are having talks from British, Mexican, Canadian, American, and Australian queer researchers. My advice to others looking to start similar programs would be to find a committed couple of individuals willing to dedicate significant time to getting your initiative off the ground. Kyle has been fabulous to work with, he is tireless in cold-emailing potential speakers, a reason we have had such diversity in our talks. Secondly, I would recommend reaching out to other queer groups, I can comfortably say that both Kyle and I would be more than happy to help promote new initiatives to our Scientific QUEERies community. It is highly likely that you will find more LGBTQ2S+ and vocal allies closer to home than you may expect. That was definitely the case for us!

**KS:** Visibility was our first objective, as we realized over a pint one day that it would be nearly impossible to find a supervisor or mentor who was queer. When you are an invisible minority, it is so easy to feel like you are alone and to be overlooked by equity, diversity, and inclusion initiatives for more visible groups. Which is why queer people’s stories are so important to hear; they are a powerful way to make the importance of equity, diversity, and inclusion feel real and necessary to cis/straight people who may not fully understand the inequalities we face. It’s because of this that we planned our seminars to be a mix of some that are only research, which shows queer people are competent, intelligent scientists who belong, and personal professional stories, which make our struggles real and tangible for allies who attend and affirm the lived experience of queer trainees and professionals who feel they are alone.

For trainees who might want to start something similar (or any passion project really): don’t wait for someone to give you permission. Scott and I decided we were going to do this, and we just did it. We cold-emailed professors across North America and invited them to something that was nothing more than a dream. A lot of people never answered the email, but for those that did, they were ecstatic that something like this was happening. You need to find the people that have buy-in to whatever you want to do and start planning. If you wait for other people or administration to tell you it’s ok, or give you resources, you will never start. It also helps to have a little bit of self-delusion when starting something like this, believe and act like you are a big deal already (a fake it until you make it situation). I know that is hard for queer people, because we often don’t believe we are worth anything, but you have got to have confidence! Lastly, don’t wait for someone else to do something important. It is a lot of work, and if you don’t have the passion and drive to make it happen, how can you expect someone else to? Do the work, don’t take no for an answer, and keep looking until you find your space and people.

**What has been your favorite seminar topic so far?**

**SC:** This is hard to narrow down. We have been lucky to host some amazing people over the past year. However, I would be biased towards presentations by Dr. Dan Gillis (University of Guelph), Dr. Jessica Ware (American Museum of Natural History) and Dr. Tara Moriarty (University of Toronto). Passionate about their research, driven to tackling systematic barriers in their respective fields, and willing to share their personal journey. Each speaker encapsulated the mission of Scientific QUEERies, to normalize our place in STEM fields, to highlight the challenges that many of us face daily, and to reiterate the unique perspectives and knowledge queer individuals bring to science.

**KS:** My favorite seminar was given by Dr. Jessica Wade from the American Museum of Natural History, who is the coolest scientist I have ever had the pleasure to meet (you can see her in a field report on the Late Show with Stephen Colbert about cicadas, check it out). She spoke with us about her research in *Odonata* (dragonflies) and working as a museum scientist and curator. But what was most compelling about her talk, was her clear and compassionate explanation of the systemic inequalities that can limit queer and people of color’s success in academia. You can watch the seminar on our website, and I really recommend that people take a look (sites.google.com/view/scientificqueeries).

*This interview was conducted by Associate Editor George Inglis*.

